# Accumulation of Azole Drugs in the Fungal Plant Pathogen *Magnaporthe oryzae* Is the Result of Facilitated Diffusion Influx

**DOI:** 10.3389/fmicb.2017.01320

**Published:** 2017-07-13

**Authors:** Brooke D. Esquivel, Theodore C. White

**Affiliations:** Division of Cell Biology and Biophysics, School of Biological Sciences, University of Missouri–Kansas City, Kansas City MO, United States

**Keywords:** *Magnaporthe*, drug import, azole antifungals, filamentous fungi, clorgyline, facilitated diffusion

## Abstract

*Magnaporthe oryzae* is an agricultural mold that causes disease in rice, resulting in devastating crop losses. Since rice is a world-wide staple food crop, infection by *M. oryzae* poses a serious global food security threat. Fungicides, including azole antifungals, are used to prevent and combat *M. oryzae* plant infections. The target of azoles is CYP51, an enzyme localized on the endoplasmic reticulum (ER) and required for fungal ergosterol biosynthesis. However, many basic drug-pathogen interactions, such as how the azole gets past the fungal cell wall and plasma membrane, and is transported to the ER, are not understood. In addition, reduced intracellular accumulation of antifungals has consistently been observed as a drug resistance mechanism in many fungal species. Studying the basic biology of drug-pathogen interactions may elucidate uncharacterized mechanisms of drug resistance and susceptibility in *M. oryzae* and potentially other related fungal pathogens. We characterized intracellular accumulation of azole drugs in *M. oryzae* using a radioactively labeled fluconazole uptake assay to gain insight on whether azoles enter the cell by passive diffusion, active transport, or facilitated diffusion. We show that azole accumulation is not ATP-dependent, nor does it rely on a pH-dependent process. Instead there is evidence for azole drug uptake in *M. oryzae* by a facilitated diffusion mechanism. The uptake system is specific for azole or azole-like compounds and can be modulated depending on cell phase and growth media. In addition, we found that co-treatment of *M. oryzae* with ‘repurposed’ clorgyline and radio-labeled fluconazole prevented energy-dependent efflux of fluconazole, resulting in an increased intracellular concentration of fluconazole in the fungal cell.

## Introduction

The fungal plant pathogen *Magnaporthe oryzae* is considered to be one of the most dangerous and destructive agricultural molds. Named after the host it infects, *M. oryzae* causes disease in rice (*Oryza sativa*) called rice blast, which can infect all parts of the rice plant causing lesions on leaves, stems, seeds, and even roots (Martin-Urdiroz et al., 2016; Yan and Talbot, 2016). Hundreds of millions of people world-wide depend on rice as a staple food crop making rice the most important grain with regard to human diet and nutrition (Harrison, 2002; Thornton and Wills, 2015). Consequently, rice blast poses a serious global food security threat. So great is the potential risk for crop failure or severely reduced crop yield due to rice blast, that *M. oryzae* has been ranked among the most significant plant pathogens (Talbot, 2003; Thornton and Wills, 2015).

Fungicides, including agricultural azoles such as propiconazole, prothioconazole, tebuconazole, and prochloraz, are used to combat rice blast (Talbot, 2003; Wilson and Talbot, 2009; Edwards and Godley, 2010; Parker et al., 2011, 2013; Kaur et al., 2012; Lehoczki-Krsjak et al., 2013, 2015). Fungicides are utilized as both seed treatment to prevent infection of the seedlings after germination, and also as foliar sprays on mature leaves to prevent infection and spread during the growing season (Couch et al., 2005; Edwards and Godley, 2010; Parker et al., 2011, 2013; Kaur et al., 2012; Lehoczki-Krsjak et al., 2013, 2015).

The target of azole antifungals is the fungal Cyp51, Lanosterol 14α-demethylase. This enzyme is a member of the cytochrome P450 superfamily of enzymes required for ergosterol biosynthesis in fungi.

Drugs that have intracellular targets, such as Cyp51 located on the endoplasmic reticulum (ER), must cross both the fungal cell wall and the plasma membrane and then be transported to its ER destination. It is not clear how azoles are able to do this. It has generally been supposed that azoles enter the cell by passive diffusion, until recent analysis of fluconazole (FLC) uptake in *C. albicans* and *A. fumigatus* by our lab argue against a passive diffusion mechanism (Mansfield et al., 2010; Esquivel et al., 2015). Identifying drug uptake mechanisms in fungi opens many new avenues for investigation in basic fungal biology, pathogenesis, and new drug design. Insights into new fungal-specific drug targets, or finding treatment strategies using combinations of existing drugs, is of great importance to control the spread of resistant fungi.

Importantly to our research, *M. oryzae* shares many characteristics associated with other notable cereal pathogens (Talbot, 2003; Wilson and Talbot, 2009). Advances in understanding the genes and mechanisms that govern the resistance and susceptibility in this species, will help advance our understanding of other related fungal diseases.

In this study, we extended our previously reported radioactively labeled fluconazole accumulation assay to measure cellular accumulation of azole drugs in *M. oryzae* and determine if azoles enter the cells by passive diffusion, active transport, or a facilitated diffusion mechanism. Consistent with our import experiments on the filamentous human fungal pathogen *A. fumigatus*, our import experiments with *M. oryzae* have shown that azole drugs do not accumulate in the fungal cell solely by passive diffusion or ATP-dependent active transport. Instead there is evidence for azole import by a facilitated diffusion mechanism such as a protein carrier or channel.

In addition to uptake analysis, we used ^3^H-FLC to measure glucose-stimulated and glucose-independent azole efflux over time from *M. oryzae*. The *M. oryzae* strain used in this study is the first plant pathogenic fungus to be fully sequenced allowing a direct examination of its genome (Dean et al., 2005). Compared to yeast species such as *Saccharomyces cerevisiae*, *Candida albicans*, *Cryptococcus neoformans*, *Candida glabrata*, and *Candida krusei*, the filamentous fungus *M. oryzae* contains an unusually high number of genes encoding predicted membrane transporters. This includes at least 50 ATP-Binding Cassette (ABC) and at least 250 Major Facilitator Superfamily (MFS) transporters (Coleman and Mylonakis, 2009; Kim et al., 2013). The majority of these transporter genes are still uncharacterized to date (Kovalchuk and Driessen, 2010; Kim et al., 2013).

Our ^3^H-FLC efflux data showed evidence for a glucose-stimulated azole efflux mechanism in *M. oryzae*, which would be consistent with an ABC superfamily transporter activity.

For a final efflux-related analysis, we evaluated the effects of combination treatment of *M. oryzae* with ^3^H-FLC and clorgyline, a drug once used as an antidepressant that has recently been shown to have efflux inhibitor properties (Youdim, 1975; Holmes et al., 2012). These results demonstrate the potential usefulness of combination drug therapies and the idea of repurposing archived compounds for alternative uses as the threat of resistance to current fungal treatments emerges.

## Materials and Methods

### Strains, Media, Materials, and Strain Preparation

The *M. oryzae* wild-type, sequenced strain 8958 (70-15), was used for all azole import experiments. *M. oryzae* cultures were routinely grown and maintained in either liquid yeast nitrogen base, ammonium sulfate and dextrose (YAD) medium (1.7 g yeast nitrogen base without amino acids or ammonium sulfate, 5 g ammonium sulfate and 20 g glucose per liter) or on oatmeal agar plates with 20 g glucose per liter, at room temperature (27°C). Conidia were harvested from 7-day old agar plate cultures by pipetting 0.01% Tween 20/water directly onto sporulating plates and loosening conidia using a sterilized glass spreader. The harvested solution was allowed to sit for 10–15 min to allow mycelia to settle, while the conidia remained suspended.

To propagate *M. oryzae*, 200 μL of the supernatant from the harvested conidial suspension was either directly inoculated into 5 ml liquid YAD media with glucose and grown in 50 mL conical tubes in a room temperature shaking at 180 rpm, or plated on oatmeal agar plates. For storage, oatmeal agar plates were overlaid with 3–5 Whatman filter paper disks before inoculating with conidial suspension. After 7–10 days of growth, filter paper disks covered with a lawn of *M. oryzae* were collected, desiccated in a Tupperware container with Drierite desiccant stones (W.A. Hammond Drierite Co. LTD. Xenia, OH, United States), and stored at -20°C.

Medium components, plastic ware, general chemicals, and unlabeled drugs used for competition were obtained from Fisher Scientific (Pittsburg, PA, United States), or Sigma–Aldrich (St. Louis, MO, United States).

### *M. oryzae E*-Test Susceptibility Testing to Common Antifungals

*E*-test strips (bioMérieux, United States) were used to test *M. oryzae* susceptibility and determine the minimum inhibitory concentration (MIC) to the antifungals fluconazole (FLC), ketoconazole (KTC), itraconazole (ITC), posaconazole (POS) voriconazole (VRC), caspofungin (CFG), amphotericin B (AMB), and 5-flucytosine (5FC). One side of the plastic *E*-test strip is calibrated with MIC values of the drug in μg/ml. The drug-gradient on the strip covers 15 twofold dilutions.

Yeast extract Peptone Dextrose, or YEPD or YPD (10 g yeast extract, 20 g peptone and 20 g glucose) plates were inoculated with a lawn of *M. oryzae* conidia and allowed to dry. A single *E*-test strip was placed on each inoculated plate and kept at 27°C for 96 h with daily monitoring. The MIC was determined based on the drug concentration on the *E*-test strip in which the zone of inhibition, or ellipse of non-growth occurred.

### Radioactively Labeled Azole Import by *M. oryzae*

Radioactively labeled FLC (^3^H-FLC), (481 GBa/mmol, 13 Ci/mmol, 1 μCi/μL; 77 μM FLC) was custom synthesized by Amersham Biosciences. The drug concentration used during the import assay was well below the MIC for the strain (*M. oryzae* FLC MIC >32 μg/ml).

To directly measure azole intracellular accumulation in the fungal cell, we used ^3^H-FLC in our drug uptake assay designed for the filamentous morphology of *M. oryzae*. Studies of plant-pathogen interactions have shown that fungal growth associated with plant disease shows biofilm-like properties including robust hyphal networks and heterogeneous fungal layers (Villa et al., 2017). Unless specifically noted otherwise, 5 ml YAD medium with 2% glucose in 50 mL conical tubes was inoculated with *M. oryzae* conidia and grown at 27°C, 180 rpm shaking, for 48 h at which point they were filamentous mycelial masses or fungal balls of hyphae, approximately 3 mm in diameter. The mycelial masses were transferred to 2 mL microcentrifuge tubes and washed by centrifugation and resuspension in fresh media three times using YNB complete (1.7 g yeast nitrogen base without amino acids or ammonium sulfate, 5 g ammonium sulfate per liter, pH 5.0) without glucose unless specifically noted.

After the washing, the fungal ball pellet was transferred to 14 mL round bottom tubes containing 1 mL of YNB for a 2 h glucose-deprived (starvation) de-energizing period. The glucose starvation was done to keep the cells in a de-energized state. The de-energized cells showed no further growth for the extent of the import assay, as determined by dry weight at the conclusion of the assay. After the glucose starvation period, the radiolabeled drug treatment reaction mixes were prepared consisting of 1 mL of YNB containing no glucose, fungal balls, and 25 μL of diluted ^3^H-FLC (freshly diluted 1/100X from stock). The resulting final ^3^H-FLC concentration is 19.25 nM (5.89 ng/ml). The *M. oryzae* strain (70–15) has an MIC of >32 μg/ml to FLC. Thus, the azole concentration used for the import assay is not expected to have any effect on cell viability.

After 24 h incubation with ^3^H-FLC, the reaction was stopped with a 5 ml stop solution (YNB +20 mM [6 mg/L] unlabeled FLC) added to each 14 mL round bottom tube sample. The samples were filtered by vacuum over pre-weighed and wetted glass fiber filters (24 mm GF/C; Whatman; Kent, United Kingdom). The samples on the filters were rinsed with 5 ml stop solution to remove non-specific, cell surface binding of ^3^H-FLC. The filters with fungal balls were either allowed to dry for 24–48 h or were baked in a drying oven for 15 min at 95°C. Each dried filter containing fungal balls was then re-weighed to obtain the dry mass of each fungal sample. The filters were finally transferred to 5 mL scintillation vials containing 3 ml of scintillation cocktail (Ecoscint XR, National Diagnostics, Atlanta, GA, United States). Radioactivity associated with the fungal sample on each filter was measured in a liquid scintillation analyzer (Beckman Coulter, LS 6500 multipurpose scintillation counter).

Radioactivity values are expressed as ^3^H-FLC drug accumulation per dry weight of the sample. Results were calculated as CPM/mg of mycelial mass. While absolute CPM values varied between experiments, relative values of import differences between samples remained consistent and repeatable.

### Screening of Factors That Modulate ^3^H-FLC Import

Further studies were done to determine how FLC import is affected by changes in the growth or drug incubation conditions.

#### Heat Inactivated Cells

Fluconazole uptake was measured in cells that had been exposed to high heat (95°C) for 30–40 min. Samples were processed according to the standard ^3^H-FLC import protocol with the exception that the heat-killing step was performed immediately prior to ^3^H-FLC treatment. Heat killed samples were treated with ^3^H-FLC and analyzed identically to the unheated, control cell samples. The results were compared with live *M. oryzae* data. Heat inactivated samples were used as a control for baseline drug accumulation and non-specific cell surface binding in all testing conditions unless otherwise noted.

#### Other Methods of Cell Inactivation

^3^H-FLC accumulation was measured after 24 h in samples that were exposed to various inactivation methods. Methods of cell inactivation included treatment with high concentrations of AMB (8 μg/ml), Qiagen RLT Lysis solution (600 μL), and CFG (16 μg/ml). Heat killing as described above was found to be the most reliable at reducing viable cell counts to less than 1%. And so this method of inactivation was used as a no-import, baseline control, although the other methods of inactivation were useful to confirm that import is reduced in inactivated cells as opposed to being an artifact of heat treatment.

#### Energy Depletion

To determine whether ^3^H-FLC import was energy-dependent, mycelial balls were washed three times from overnight media and resuspended in glucose-free YNB media. Samples were glucose starved for 2 h in this media and considered to be de-energized after this time. Following the de-energization period, the cells were treated with ^3^H-FLC for 24 h in glucose-depleted media and their ^3^H-FLC accumulation was compared to cells that were treated with ^3^H-FLC for 24 h in media containing 2% glucose.

#### pH

Cells were incubated with ^3^H-FLC in YNB without supplementation (pH 5), with no pH adjustment, or with YNB medium adjusted to pH 4 or 7 with a 0.1 M citric acid and 0.2 M Na_2_HPO_4_ mixed buffer stock solution. Increasing the ratio of the citric acid to Na_2_HPO_4_ to lower pH and increasing the ratio of Na_2_HPO_4_ to citric acid solution to increase pH. All samples were processed after 24 h as described above.

#### Growth Media

*Magnaporthe oryzae* liquid cultures were grown from conidia to mature mycelial masses (48 h) in either Complete Supplemental Medium or CSM (0.75 g CSM, 1.7 g yeast nitrogen base without amino acids or ammonium sulfate, 5 g ammonium sulfate, 20 g glucose per liter); YAD Medium (1.7 g yeast nitrogen base without amino acids or ammonium sulfate, 5 g ammonium sulfate and 20 g glucose per liter); or YPD as described above. The mycelial masses were then washed and treated with ^3^H-FLC in YNB for 24 h as described for the standard import assay.

CSM is a Complete Supplemental Mixture that is considered synthetic defined, complete medium, containing the chemically defined components Yeast Nitrogen Base (YNB), Ammonium Sulfate (AS) and a carbon source, glucose. It also contains a complete amino acid supplement mixture and so is non-selective.

Yeast nitrogen base, ammonium sulfate and dextrose is considered a synthetic defined, minimal medium, containing the chemically defined components YNB, AS and glucose, but without any supplemented amino acids so only prototroph strains can grow in this media.

Yeast extract Peptone Dextrose, also called YEPD, is considered an undefined, non-selective, rich medium. YPD contains Yeast extract, a complex nutrient base derived from killed yeast cells; Peptone, an enzymatic digest of animal protein that contains nitrogen and a high peptone and amino acid content, and glucose. This medium provides an excess of amino acids, nucleotide precursors, vitamins, and essential metabolites.

#### Exponential vs. Stationary Phase Uptake of ^3^H-FLC

Conidia were grown at 27°C in a shaking incubator at 180 rpm in YAD complete media with glucose for either the standard 48 h (exponentially growing) or for 72 h or 96 h (stationary phase). These 2, 3, or 4 days old fungal balls were then treated with ^3^H-FLC for 24 h as described previously.

#### Competitive Inhibition of Azole Import

To determine if azoles enter the cell by a specific or non-specific protein carrier, or by passive diffusion, we treated *M. oryzae* fungal balls with ^3^H-FLC as well as co-treatment with unlabeled compounds that could be potential competitive inhibitors. Samples were processed as described above with our ^3^H-FLC import assay, with the additional compound added to the sample at 1.95 μM (100X molar excess of the labeled FLC). ^3^H-FLC uptake was measured as usual after 24 h incubation with ^3^H-FLC and competitor.

Decreased ^3^H-FLC uptake in the presence of unlabeled FLC as well as other unlabeled competitor suggests that both drugs use the same transporter. Non-radiolabeled drugs used for import competition experiments included: amphotericin B (AMB), caspofungin (CFG), fenpropimorph (FEN), fluconazole (FLC), itraconazole (ITC), ketoconazole (KTC), metconazole (MET), tebuconazole (TBZ), terbinafine (TRB), and 1-triphenylmethyl imidazole (1-TRI).

### Azole Efflux from *M. oryzae*

#### Efflux Kinetics

*Magnaporthe oryzae* mycelial masses of hyphal cells were preloaded with ^3^H-FLC by incubating samples with the radiolabeled FLC at 19.5 nM in the absence of glucose for 24 h to allow maximum intracellular accumulation with no energy-dependent glucose. After the 24 h incubation, the mycelial masses were washed and resuspended into fresh YNB media, and the amount of labeled drug remaining in the cells over time was determined at 4, 8, and 16 h. Efflux was evaluated in both glucose-energized (2% glucose) and de-energized (glucose-starved) cells.

#### Efflux Inhibition by Clorgyline

To test the effect of Clorgyline as a possible inhibitor of energy-dependent efflux in *M. oryzae*, we treated energized cells (2% glucose, efflux active) with either ^3^H-FLC alone (19.25 nM) or ^3^H-FLC with clorgyline [*N*-Methyl-*N*-propargyl-3-(2,4-dichlorophenoxy)propylamine hydrochloride] (233 μM) and compared the results to ^3^H-FLC treatment in de-energized (glucose-starved, efflux inactive) cells. ^3^H-FLC accumulation was measured in all conditions after 20 h.

#### Statistical Analysis

Differences between sets of samples were evaluated by an unpaired two-tailed Student’s *t*-test. A *P*-value of < 0.05 was considered significant.

## Results

### *M. oryzae* Minimum Inhibitory Concentrations (MIC) to Common Antifungals

**FIGURE 1:**
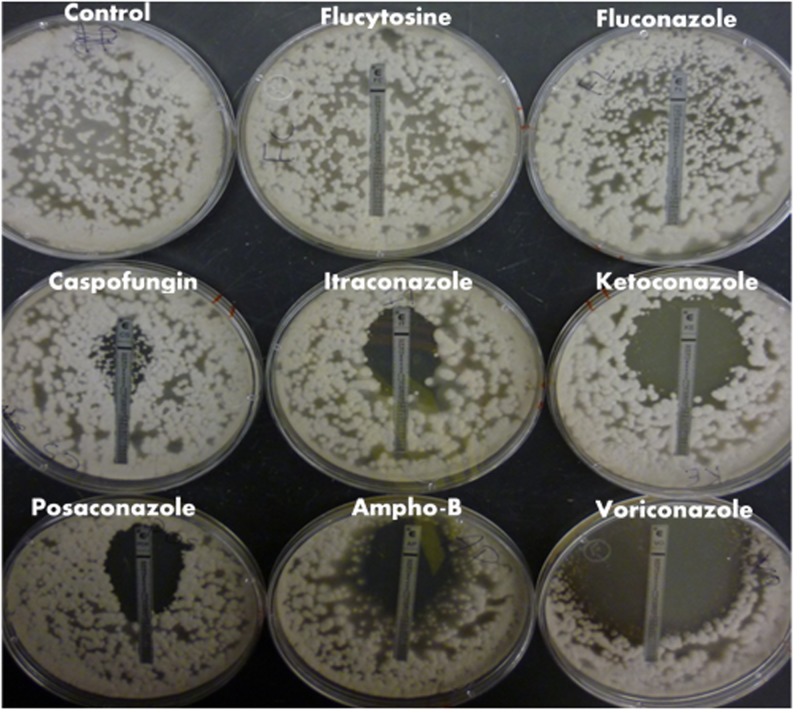
*Magnaporthe oryzae* drug susceptibility to common classes of medical antifungals. *E*-test strips were used to test *M. oryzae* susceptibility and determine the MIC to common antifungals belonging to different classes of drug. Plates were allowed to grow at 27°C for 96 h. The MIC was determined based on the ellipse of non-growth intersecting with the drug gradient marked on the test strip.

*E*-test strips were used to establish *M. oryzae* susceptibility to common medical antifungals belonging to different classes of drugs and determine if a study using medical azoles was relevant in *M. oryzae*. The MICs were reported in **Figure 1** and **Table 1**. The drugs tested included the medical azoles FLC, ITC, KTC, POS, and VRC, the echinocandin CFG, the polyene AMB, and the RNA and DNA synthesis inhibitor 5FC.

**Table 1:** *M. oryzae* minimum inhibitory concentrations (MICs) by *E*-test.

Drug	μg/ml
5FC	>32
FLC	>32
CFG	>32
ITC	0.5
KTC	0.5
POS	0.38
AMB	0.25
VRC	0.06


*Magnaporthe oryzae* did not show susceptibility to 5FC or FLC at the drug concentrations tested, and although there was a ‘phantom’ zone of clearing around the CFG *E*-test, there were enough CFG-resistant colonies in the clearing for this strain to be interpreted as resistant at the concentrations tested. There was measurable susceptibility of *M. oryzae* to the other drugs tested (**Figure 1** and **Table 1**).

### Factors That Modulate ^3^H-FLC Import

Recently, radioactively labeled drugs were used to analyze azole uptake in the human pathogenic yeast *Candida albicans* and *Cryptococcus neoformans*, the model yeast *Saccharomyces cerevisiae*, and the human pathogenic mold *A. fumigatus* (Mansfield et al., 2010; Esquivel et al., 2015). Here we have adapted the assay used in the previous studies for an initial characterization of azole uptake and efflux in the plant pathogenic mold *M. oryzae*. Radioactive FLC is used in these import studies because of its availability. While the MIC analysis (**Figure 1** and **Table 1**) indicates that FLC is not effective against *M. oryzae*, the results presented indicate that the drug is imported into the cell as well as other azoles.

#### ^3^H-FLC Import in Inactivated Cells

**FIGURE 2:**
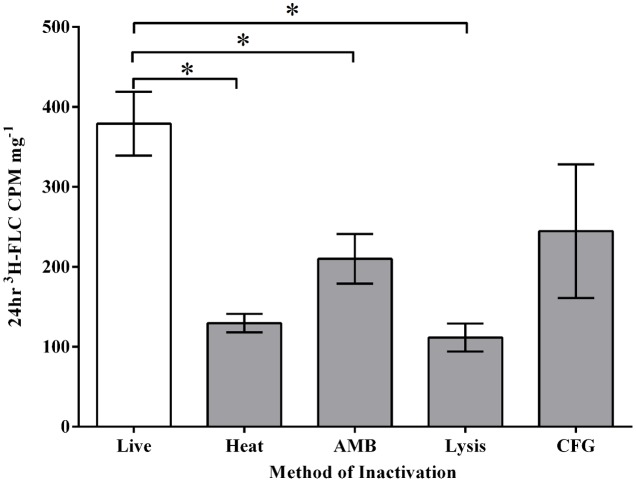
*Magnaporthe oryzae* inactivation methods before ^3^H-FLC treatment. ^3^H-FLC accumulation was measured after 24 h in samples that were exposed to various inactivation methods. (Live): no inactivation, (HK): 95°C for 30 min, (AMB): 8 μg/ml of amphotericin B, (Lysis): 600 μL Qiagen RLT buffer, (CFG): 16 μg/ml of caspofungin. Error bars represent standard deviation for each condition. Asterisk indicates a statistical significance of *P* < 0.05.

Labeled FLC was used in the import assay to characterize azole import. Cell inactivation by heat, AMB treatment, and lysis solution each reduced azole import significantly (**Figure 2**), suggesting drug uptake and accumulation requires the maintenance of an intact and functional cell membrane. Inactivation by high heat incubation before ^3^H-FLC treatment was the chosen method for creating a baseline control sample because heat inactivation reliably and reproducibly reduced intracellular ^3^H-FLC to very low radioactive counts. Heat inactivation was therefore used in subsequent experiments as a measure of background, or non-specific drug binding.

The reduced ^3^H-FLC import due to heavy AMB treatment also gives support to the idea that the uptake of FLC is at least partially dependent on the plasma membrane, perhaps via a protein channel or cell membrane transporter. Disruption of the membrane by AMB treatment above MIC levels (8 μg/ml) partially but significantly inhibits azole uptake.

Caspofungin treatment at 16 μg/ml reduced FLC uptake into the cell but not significantly, suggesting that cell wall disruption does not influence azole import in the way that plasma membrane disruption does. However, the CFG-treated cells did show high import variability and a trend toward reduced azole import so the role of the cell wall for azole import remains to be elucidated.

#### Energy-Dependent Active Transport

**FIGURE 3:**
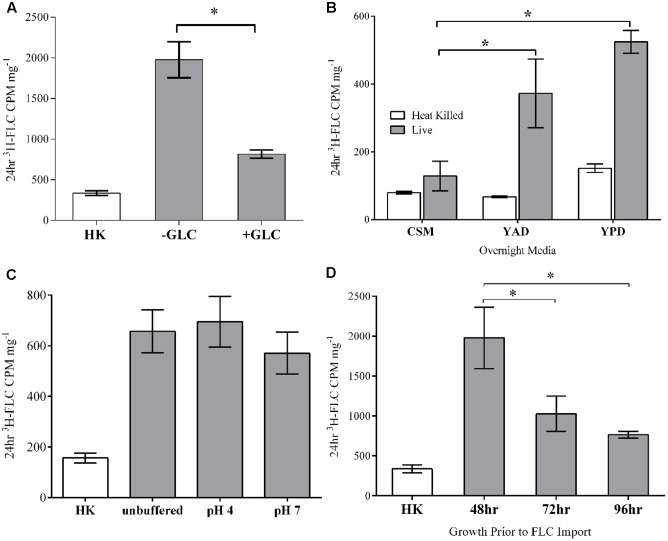
Effects of glucose, media, pH and cell phase on FLC import. **(A)** Glucose: Cells were de-energized by glucose-depleted media (–GLC) compared to cells in the presence of glucose (+GLC) and measured for FLC accumulation. **(B)** Growth Media: Cells were grown from conidia to mycelial masses for 48 h in either CSM complete, YAD, or YPD media. **(C)** pH: ^3^H-FLC was imported into *M. oryzae* in unbuffered media or at pH 4 and 7 in 100 mM citric acid buffers. There were no statistically significant differences between import at the three conditions. **(D)** Phase: Cells were grown in shaking liquid media for 48, 72, or 96 h and then treated with ^3^H-FLC for 24 h. For each panel: Error bars represent standard deviation for each condition. Asterisks indicate a statistical significance of *P* < 0.05 between the two conditions connected by brackets. HK, heat killed control. Statistical differences to HK are not shown.

To determine if *M. oryzae* actively takes up ^3^H-FLC by an energy-dependent active transport mechanism, we analyzed ^3^H-FLC accumulation in the presence or absence of glucose as an energy source for ATP-coupled transporters (ABC transporters). The cells for each sample were first de-energized by glucose starvation in glucose-depleted media prior to ^3^H-FLC incubation to deplete cellular ATP. The ^3^H-FLC incubation period was then carried out in either glucose replete or glucose deplete conditions and the results were compared for quantifying the effect of energy-dependence during import (**Figure 3A**).

^3^H-FLC accumulated in both de-energized (glucose-starved) and energized (glucose-replete) cells, with a significantly higher intracellular concentration in the de-energized cells. A mechanism of drug transport across the cell membrane that does *not* require energy, such as the de-energized sample, argues against ATP-dependent active import of FLC in *M. oryzae.*

The significant reduction of intracellular ^3^H-FLC concentration in glucose-energized samples, compared to de-energized samples, is most likely the result of activation of ATP-dependent efflux pumps for which azoles have been identified as substrates in many fungi. For subsequent experiments, we used de-energized (glucose-starved) cells to focus solely on drug uptake and eliminate efflux mechanisms.

#### Growth Media

*Magnaporthe oryzae* was grown from conidia to mature mycelial masses in either CSM, YAD, or YPD media and then all samples were washed and resuspended in YNB medium and the ^3^H-FLC accumulation assay was started. ^3^H-FLC accumulation was compared between the samples that had previously been grown in the different media (**Figure 3B**).

Cells that were grown from conidia to mycelial balls in CSM took up the least amount of ^3^H-FLC after 24 h treatment in the assay. Cells initially grown to mycelial balls in YPD media took up the most ^3^H-FLCin the assay. However, the baseline control was also slightly higher in the YPD-grown cells as well. Cells initially grown in YAD took up an intermediate level of ^3^H-FLC in the assay, but surprisingly, uptake was more similar to cells from YPD media compared to the CSM. Both YAD and YPD-grown samples had significantly higher import compared to CSM-grown cells, which had near baseline levels of import, even though the ^3^H-FLC treatment and import assay for all samples was performed identically.

The data suggest some alteration to, or adaptation of, the cell acquired from the different media that affect FLC uptake and accumulation. The mechanism behind this variation is not known and could be a complex mixture of differences in protein synthesis, lipid storage, transcriptional activity, and other metabolic activities. An examination of the effects of different media on cell wall and cell membrane composition and function would be useful to better understand this phenomenon.

Fungal cell environmental adaptations that prevent or enhance azole uptake would be an important aspect for drug resistance analysis. This differential azole uptake between the samples argues against passive diffusion entry into the cell.

#### pH

To determine if ^3^H-FLC import is pH dependent or affected by a proton gradient, we measured ^3^H-FLC accumulation in unbuffered YNB media, or at pH 4 or 7 using a 0.1 M citric acid and 0.2 M Na_2_HPO_4_ mixed buffer (**Figure 3C**). There was significant ^3^H-FLC accumulation at all pHs tested, but with no statistically significant difference between the different pHs. This indicates that ^3^H-FLC import is not pH dependent, or at least not constrained to a specific pH.

#### Stationary vs. Exponential Cells

Samples grown for the standard 48 h before being incubated with ^3^H-FLC for uptake analysis were compared to samples grown for 72 or 96 h before drug treatment. **Figure 3D** shows that the exponentially growing (48 h) samples accumulated significantly more ^3^H-FLC than the stationary phase (72 and 96 h) samples. The fact that there is differential uptake of ^3^H-FLC between the samples of different age, stage of hyphal growth, or metabolic activity, would not be observed with passive diffusion and provides evidence for a facilitated transport mechanism of FLC import.

### Competition for ^3^H-FLC Import in *M. oryzae* with Azoles and Other Compounds

**FIGURE 4:**
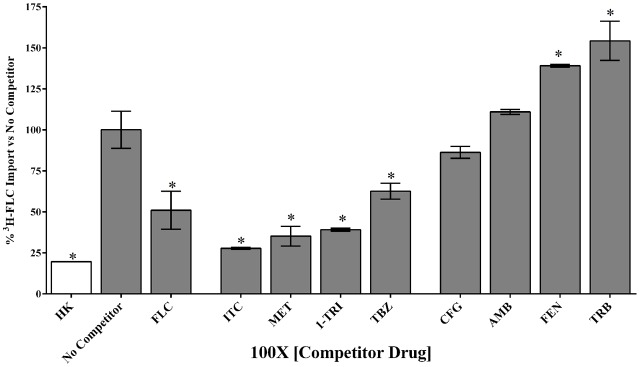
Competition for ^3^H-FLC import in *M. oryzae.* Unlabeled compounds were tested for competition at 1.95 μM (100x molar excess to ^3^H-FLC) during simultaneous treatment with ^3^H-FLC. ^3^H-FLC accumulation was measured after 24 h incubation with competitors. Asterisks indicate compounds that showed significantly different ^3^H-FLC accumulation of *P* < 0.05 from the No Competitor control. Error bars represent standard deviation of biological triplicates for each condition. Drug abbreviations are described in section “Materials and Methods.”

To determine whether all azoles use the same transporter or family of transporters in *M. oryzae*, non- labeled azoles were tested for competition against labeled FLC (**Figure 4** and **Tables 2**, **3**). The concentration of all competitors was 1.95 μM (100x molar excess to ^3^H-FLC), which is well below inhibitory drug treatment concentrations.

**Table 2:** Compounds that compete at 100X molar excess with FLC for import in *M. oryzae.*

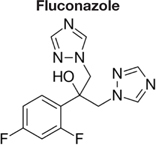	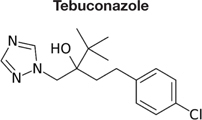
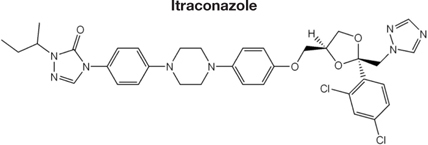	
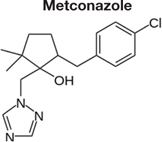	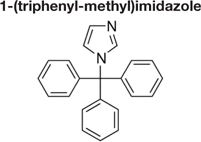


**Table 3:** Compounds that do not compete at 100X molar excess with FLC for import in *M. oryzae.*

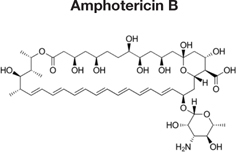	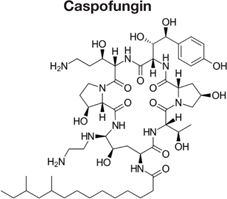
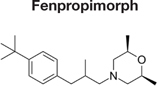	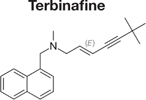


Uptake can clearly be modulated by a variety of pharmacologic agents that can be described as compounds that competed with FLC for uptake, compounds that caused no effect on FLC uptake, and compounds that might stimulate FLC uptake.

Competitive inhibitors, indicated by a significant reduction of the ^3^H-FLC accumulation to baseline levels, included the medically important azoles (FLC, ITC) as well as agriculturally important azoles (MET, TBZ). 1-TRI, a clotrimazole analog, also competed with FLC for import. Competitive inhibition of ^3^H-FLC import by structural analogs- namely, other azoles is a strong argument against passive diffusion and in favor of facilitated diffusion, possibly via a protein channel.

Other compounds were also tested for competition against ^3^H-FLC uptake including common non-azole antifungals (**Figure 4** and **Tables 2**, **3**). Non-azole compounds such as AMB and CFG, did not compete with ^3^H-FLC for import into *M. oryzae.* The antifungals TRB and FEN also did not compete with ^3^H-FLC uptake and may even stimulate ^3^H-FLC accumulation (**Figure 4**).

The results of these competition experiments indicate transport specificity for certain chemical structures. The structure of FLC and the compounds tested in this assay are shown in **Tables 2**, **3**. FLC has two 5-membered triazole rings containing 3 nitrogen atoms, and a 6-member halogenated benzene ring. Previous screens of moieties important for azole import in *C. albicans* and *A. fumigatus* are consistent with this result in *M. oryzae* (37, 38). Taken together, analyses in *C. albicans*, *A. fumigatus*, and now *M. oryzae* suggests that to compete for FLC import, a compound requires a 5-membered ring with two (imidazole) or three (triazole) nitrogen atoms, in addition to a halogenated 6-membered ring with the halogen in position 1 or 3, but not necessarily both positions (Mansfield et al., 2010; Esquivel et al., 2015). The only exception to this has been the competition of 1-TRI, which has a five membered imidazole ring as well as three 6 membered rings, but is not halogenated.

### Efflux of ^3^H-FLC from Preloaded Cells

**FIGURE 5:**
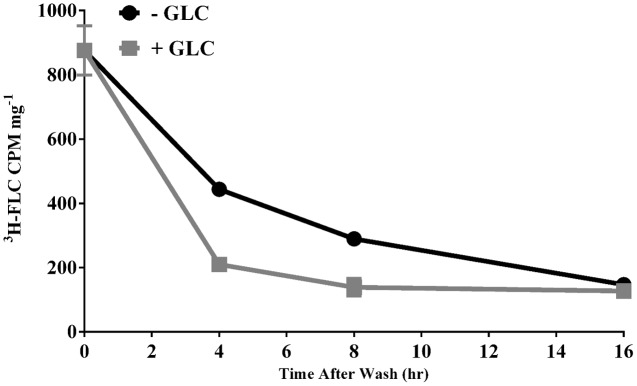
Efflux of ^3^H-FLC from preloaded cells. *M. oryzae* mycelial cells were preloaded with 19.25 nM ^3^H-FLC for 24 h. The cells were washed with YNB and placed in either de-energized (glucose free) (solid black line with circles) or a 2% glucose-energized (gray line with squares) media for 4, 8, and 16 h to measure the efflux of ^3^H-FLC. Error bars represent standard deviation for each condition. Most error bars are hidden by the symbols.

Drug accumulation in a fungal cell can be thought of as the net effect of uptake and efflux mechanisms. The conditions of our ^3^H-FLC **import** assay remove the energy dependent efflux component so that we can focus on drug entry into the cell. Conversely, **Figure 5** demonstrates **efflux** of FLC from *M. oryzae* cells. The cells were preloaded with ^3^H-FLC at 19.5 nM for 24 h following the standard import assay protocol and then were washed and resuspended in fresh media with and without glucose so as to see the effect of energy-dependent efflux on intracellular ^3^H-FLC concentration. The amount of labeled drug associated with the cells was determined at 4, 8, and 16 h after being transferred to fresh media. Efflux was evaluated in both glucose-energized (gray line with squares) and de-energized (glucose starved) (black line with circles) cells.

The difference in rate of efflux between energized and de-energized cells at 4 and 8 h was significantly different (**Figure 5**). By 16 h, most of the ^3^H-FLC was exported from the cells in both energized and de-energized conditions. The samples incubated in the presence of glucose show faster export of labeled drug, consistent with the idea that membrane efflux pumps require energy and that glucose starvation limited the energy and thus the efflux pump activity.

### Clorgyline as a Potential Efflux Inhibitor

Development of a new drug structure and a new mechanism of action would be a valuable addition to our antifungal repertoire; particularly against resistant fungal infections. However, the cost, time and knowledge required to design and develop a brand new compound, as well as testing and approval required before use in plants, animals or humans is very prohibitive. An alternative approach would be the repurposing, or modification of existing drugs, to expedite the drug development process.

Clorgyline is a monoamine oxidase-A inhibitor (MAOI) that has been used for decades as a clinical antidepressant (Fowler et al., 1981; Tipton, 1997). As with many MAOIs, side effects include a hypersensitivity to tyramine-containing foods (especially cheese), and this negative dietary interaction has led to improvements and drug alternatives so that clorgyline use has markedly declined for treatment of depressive illnesses (Fowler et al., 1981; Tipton, 1997).

However, clorgyline remains in the Library of Pharmacologically Active Compounds (LOPAC), which is a collection of compounds, marketed drugs and pharmaceutically relevant structures annotated with biological activities. These compounds can be purchased pre-solubilized, normalized, easily resupplied, and ready-to-use for studies on repurposing applications (Sigma–Aldrich).

**FIGURE 6:**
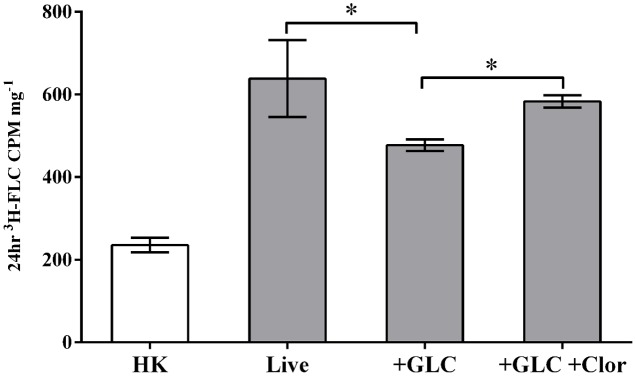
Clorgyline as a potential efflux inhibitor. Energized cells (2% glucose, efflux active) were treated with either ^3^H-FLC alone (+GLC) or ^3^H-FLC with clorgyline (233 μM) (+GLC +Clor) and the results were compared to ^3^H-FLC treatment in de-energized (glucose-starved, efflux inactive) cells (Live). ^3^H-FLC accumulation was measured after 20 h in all conditions. Asterisks indicate ^3^H-FLC accumulation that is significantly different (*P* < 0.05) between the two conditions connected by brackets. There was no significant difference in ^3^H-FLC accumulation between Live and +GLC +Clor. ^3^H-FLC accumulation in HK samples was significantly lower than all other conditions. Error bars represent standard deviation of biological triplicates for each condition.

Clorgyline has recently been identified in a screen as an inhibitor of two *C. albicans* ABC efflux pumps, CaCdr1p and CaCdr2p, as well as reversing FLC resistance in *S. cerevisiae* strains expressing ABC transporters (Holmes et al., 2012). To examine the effect of clorgyline as a possible inhibitor of energy-dependent efflux in *M. oryzae*, we treated energized (efflux active) cells with either ^3^H-FLC alone or ^3^H-FLC with clorgyline and compared the results to ^3^H-FLC treatment in de-energized (efflux inactive) cells (**Figure 6**).

Consistent with **Figure 3A**, ^3^H-FLC uptake was observed in both de-energized and energized cells, with the energized cells showing significantly reduced intracellular ^3^H-FLC concentration compared to de-energized cells (**Figure 6**). The reduced azole accumulation in the energized cells is most likely the result of activation of energy-dependent efflux pumps transporting the ^3^H-FLC out of the cell. However, when the energized, efflux-active cells were treated with clorgyline, there was significantly increased intracellular ^3^H-FLC accumulation, indicating energy-dependent efflux was at least partially prevented by the addition of clorgyline.

## Discussion

There is a continual emergence of fungicide-resistant pathogens in clinical and agricultural isolates (Fleming et al., 2002). Understanding even the most basic cellular processes and epidemiology of these isolates is imperative to prevent their spread, determine treatment and prevention strategies, optimize future drug design, and to predict future evolution of resistance (Ashkani et al., 2011).

Many fungi with intrinsic resistance to antifungal agents already exist in our environment (36). We illustrated this with *E*-test susceptibility testing of *M. oryzae*, which displayed FLC and CFG resistance (**Figure 1**). Most filamentous fungal species and molds are intrinsically resistant to FLC since they possess multiple target CYP51 paralogs: two in *M. oryzae*, *A. fumigatus*, and *A. nidulans*, and three in *A. flavus* and species of *Fusarium* (Lepesheva and Waterman, 2007). This CYP51 redundancy allows for slight changes to occur in the active site of one or all Cyp51 copies that affects the binding affinity to azoles (Hawkins et al., 2014). The structure of FLC in particular allows multiple Cyp51 active site binding conformations that are weak or transitory so that there is incomplete inhibition of the Cyp51 target enzyme compared to other azoles that have stronger Cyp51 binding affinity (Fleming et al., 2002; Mellado et al., 2005; Cools et al., 2010; Warrilow et al., 2010; Parker et al., 2011; Yan et al., 2011; Hawkins et al., 2014).

Regardless, the *E*-tests confirmed that even azole drugs used to treat human fungal pathogens are taken up by the plant pathogen *M. oryzae* as evidence by growth inhibition seen with the medical azoles ITC, KTC, POS, and VRC. Overall, the susceptibility and resistance patterns illustrated by the *E*-tests suggest a common mechanism of action of azoles on all fungal species, including the requirement for entry into the cell, passing through the cell wall and plasma membrane. This may be the first time reporting MICs of medical antifungals on a distinctly plant pathogen.

In this work, we have begun to analyze potential molecular mechanisms of azole drug resistance in the filamentous plant pathogen *M. oryzae* by characterizing azole import into the fungal cell under a variety of environmental conditions, as well as azole efflux from the cell, using radioactively labeled FLC. Azoles must enter the fungal cell in order to inhibit the intracellular Cyp51 target enzyme. Therefore reduced or modified drug import may help to explain why some pathogenic fungi are more resistant to azoles than others. Our assay can be used to compare drug import in agricultural, medical and other pathogenic fungi.

Our experiments thus far have demonstrated that azole entry into the fungal cell is not solely by a passive diffusion mechanism. There may be some baseline level of azole passive diffusion into the cell but our evidence suggests azoles import into *M. oryzae* is more substantially through a plasma membrane-localized protein channel or carrier. Likewise, there may be a certain amount of azole import due to uncharacterized ATP-dependent importers that was masked by the high activity of ATP-dependent efflux transporters. Drug uptake was observed in de-energized cells (**Figure 3A**). However, media that contained glucose showed reduced final drug accumulation levels, presumably due to activation of glucose-dependent efflux pumps.

Our data strongly support the idea of azole entry into the cell by facilitated diffusion via a membrane protein channel or carrier that recognizes a specific moiety found in azole drugs. This finding is in agreement with studies on the human pathogenic fungi *C. albicans*, *C. neoformans*, and *A. fumigatus* as well as the model yeast *S. cerevisiae* (Mansfield et al., 2010; Esquivel et al., 2015).

Import of azoles did not require a proton gradient as no change was observed in uptake over a range of buffered pHs (**Figure 3C**). There was a trend toward alkaline sensitivity for drug uptake as seen by a decrease in ^3^H-FLC uptake in samples at pH 7 media. However, a deficiency in cell growth was observed in *M. oryzae* cells at pH 7, so import at this pH may be affected by other cellular factors directly or indirectly related to pH and proton gradients.

We did find significant differences in drug accumulation in *M. oryzae* depending on the growth media used, as shown in **Figure 3B**. Environmental adaptations that prevent or enhance azole uptake is an important aspect for drug resistance and treatment analysis. Based on these import results and a comparison of the components of these three medias, it is difficult to identify a single factor that would cause such a dramatic difference in uptake between CSM compared to YAD and YPD medias. The cell adaptations to the different media that affect FLC uptake is probably a complex mixture of differences in protein synthesis, lipid storage, transcriptional activity, and other metabolic activities that may alter the cell membrane composition. The altered azole uptake between the samples argues against passive diffusion entry into the cell, in which case one would expect only minimal reduction or increase in drug accumulation between the samples.

A comparison of drug import in exponential vs. post-exponentially growing cells (**Figure 3D**) shows that fluconazole accumulation is cell phase dependent. Drug accumulation in the older, post-exponential or stationary growing cells is dramatically reduced compared to the exponentially growing cells. This is consistent with major differences in cell metabolic activity when comparing exponentially growing cells and stationary phase cells, including changes to transcription, protein translation, modifications, and secretion, membrane maintenance, and other vital cell processes.

Exponentially growing cells are considered more active and responsive, while stationary phase cells shift to a period of maintenance and conservation (Werner-Washburne et al., 1993). It has been shown for some drugs, that proliferating cells are more sensitive than those cells in a steady state, so an increase in intracellular drug concentrations in exponential phase cells compared to stationary cells would correspond with those observations (43). This could be responsible for the decrease in azole uptake in exponential phase cell growth. The differential uptake of azoles into exponential cell phase is evidence against passive diffusion in which there would be no cell phase dependency.

Strong evidence for a saturable protein carrier is illustrated by competitive inhibition of ^3^H-FLC uptake by other azoles (**Figure 4**). ^3^H-FLC import was significantly inhibited by simultaneous treatment with an excess of unlabeled azoles. However, other antifungal drugs did not compete for import into the cell and may even stimulate uptake of azoles. This indicates substrate specificity for moieties found in the azole structure. The structure of competitive inhibitors and drugs that did not compete for import are shown in **Tables 2**, **3**, respectively.

It is important to understand how different compounds are taken into the cell for the purposes of dosing and increasing treatment effectiveness, and also for combination therapy considerations. A combination of drugs that have the same mode of action but different mechanisms of uptake, may have an effect on synergy of the drugs. Antagonism may also be a result of competition for import into the cell.

Taking the ^3^H-FLC assay in another direction, we used radioactively labeled FLC to measure azole release from azole-preloaded hyphal cells. Our evidence indicates that the efflux of azoles is stimulated by energy (**Figure 5**) potentially via ABC efflux transporters, which suggests there are distinct transporters for influx and efflux of azoles, as opposed to a single transporter which functions in both directions.

The ABC superfamily of efflux pumps are well characterized for their ability to carry a broad range of substrates including, but not limited to, antifungal drugs across biological membranes (Decottignies et al., 1994; Holland and Blight, 1999; Wilkens, 2015). Resistance to commonly used antifungals has frequently been shown to develop due to overexpression or increased activity of ABC transporters in human fungal pathogens (Cannon et al., 2009; Sanglard, 2016). More recently, ABC transporters are being recognized for their role in pathogenicity, virulence, stress tolerance, and drug resistance in *M. oryzae* and other plant pathogens (Urban et al., 1999; Sun et al., 2006; Gupta and Chattoo, 2008; Price et al., 2015).

Much of the efflux of ^3^H-FLC from *M. oryzae* in glucose-stimulated and glucose-independent conditions occurred before the 4 h time point and so earlier time points would be useful for an in-depth analysis of efflux kinetics in *M. oryzae* (**Figure 5**).

The addition of clorgyline, a compound in the LOPAC collection and potential inhibitor of energy-dependent efflux, showed reduced efflux of ^3^H-FLC from energized *M. oryzae* cells (**Figure 6**). The ability of a compound to increase antifungal drug accumulation in the cell or prevent the rapid efflux of a drug makes it an ideal candidate to be considered for a synergistic combination therapy. A more thorough examination of clorgyline as an efflux inhibitor is needed, but our and others’ preliminary data suggest that clorgyline, or similar compounds, could potentially be used as combination therapy in either agriculture or clinical practice. The notion of considering medically now-outdated drugs for use as agricultural treatments unlocks many new possibilities. It might be more practical to reconsider ‘failed’ human treatment strategies, whether due to pharmacologic properties of absorption, distribution, adherence, etc. in humans, for use in plant fungal pathogens. Repurposing compounds from the LOPAC collection would shave years off the drug development process as these compounds have previously been well characterized, synthesized, stabilized, and near optimized.

## Author Contributions

Design of the study: TW and BE. Experimental work: BE. Data analysis and interpretation: TW and BE. Writing the manuscript: BE. Final approval: TW and BE.

## Conflict of Interest Statement

The authors declare that the research was conducted in the absence of any commercial or financial relationships that could be construed as a potential conflict of interest.
